# Sexual inactivity and sexual satisfaction among women living with HIV in Canada in the context of growing social, legal and public health surveillance

**DOI:** 10.7448/IAS.18.6.20284

**Published:** 2015-12-01

**Authors:** Angela Kaida, Allison Carter, Alexandra de Pokomandy, Sophie Patterson, Karène Proulx-Boucher, Adriana Nohpal, Paul Sereda, Guillaume Colley, Nadia O'Brien, Jamie Thomas-Pavanel, Kerrigan Beaver, Valerie J Nicholson, Wangari Tharao, Mylène Fernet, Joanne Otis, Robert S Hogg, Mona Loutfy, on behalf of the CHIWOS Research Team

**Affiliations:** 1 Faculty of Health Sciences, Simon Fraser University, Burnaby, British Columbia, Canada; 2 British Columbia Centre for Excellence in HIV/AIDS, Vancouver, British Columbia, Canada; 3 Chronic Viral Illness Service, McGill University Health Centre, Montreal, Quebec, Canada; 4 Department of Family Medicine, McGill University, Montreal, Quebec, Canada; 5 Women's College Research Institute, Women's College Hospital, Toronto, Ontario, Canada; 6 Women's Health in Women's Hands Community Health Centre, Toronto, Ontario, Canada; 7 Département de Sexologie, Université du Québec à Montréal, Montréal, Quebec, Canada; 8 Department of Medicine and Institute of Health Policy, Management and Evaluation, University of Toronto, Toronto, Ontario, Canada

**Keywords:** HIV, women, Canada, sexual and reproductive health, sexual abstinence, sexual satisfaction, community-based research, antiretroviral therapy, CHIWOS

## Abstract

**Introduction:**

Women represent nearly one-quarter of the 71,300 people living with HIV in Canada. Within a context of widespread HIV-related stigma and discrimination and on-going risks to HIV disclosure, little is known about the influence of growing social, legal and public health surveillance of HIV on sexual activity and satisfaction of women living with HIV (WLWH).

**Methods:**

We analyzed baseline cross-sectional survey data for WLWH (≥16 years, self-identifying as women) enrolled in the Canadian HIV Women's Sexual and Reproductive Health Cohort Study (CHIWOS), a multisite, longitudinal, community-based research study in British Columbia (BC), Ontario (ON) and Quebec (QC). Sexual inactivity was defined as no consensual sex (oral or penetrative) in the prior six months, excluding recently postpartum women (≤6 months). Satisfaction was assessed using an item from the Sexual Satisfaction Scale for Women. Multivariable logistic regression analysis examined independent correlates of sexual inactivity.

**Results:**

Of 1213 participants (26% BC, 50% ON, 24% QC), median age was 43 years (IQR: 35, 50). 23% identified as Aboriginal, 28% as African, Caribbean and Black, 41% as White and 8% as other ethnicities. Heterosexual orientation was reported by 87% of participants and LGBTQ by 13%. In total, 82% were currently taking antiretroviral therapy (ART), and 77% reported an undetectable viral load (VL<40 copies/mL). Overall, 49% were sexually inactive and 64% reported being satisfied with their current sex lives, including 49% of sexually inactive and 79% of sexually active women (*p*<0.001). Sexually inactive women had significantly higher odds of being older (AOR=1.06 per year increase; 95% CI=1.05–1.08), not being in a marital or committed relationship (AOR=4.34; 95% CI=3.13–5.88), having an annual household income below $20,000 CAD (AOR: 1.44; 95% CI=1.08–1.92), and reporting high (vs. low) HIV-related stigma (AOR=1.81; 95% CI=1.09–3.03). No independent association was found with ART use or undetectable VL.

**Conclusions:**

Approximately half of WLWH in this study reported being sexually inactive. Associations with sexual dissatisfaction and high HIV-related stigma suggest that WLWH face challenges navigating healthy and satisfying sexual lives, despite good HIV treatment outcomes. As half of sexually inactive women reported being satisfied with their sex lives, additional research is required to determine whether WLWH are deliberately choosing abstinence as a means of resisting surveillance and disclosure expectations associated with sexual activity. Findings underscore a need for interventions to de-stigmatize HIV, support safe disclosure and re-appropriate the sexual rights of WLWH.

## Introduction

Globally, women account for over half of all adults living with HIV [1]. In Canada, approximately one-quarter of the 71,300 people living with HIV (PLWH) are women, nearly double the proportion observed in 1999 (12%) [2]. With early and sustained use of antiretroviral therapy (ART), women living with HIV (WLWH) are living longer and healthier lives [3–5] with improved sexual and reproductive options accompanying lowered risks of sexual and perinatal HIV transmission [6–8]. This altered landscape of HIV risk has re-ignited global discourse regarding the need for a rights-based approach to sexual health [9–12]. Sexual health research and programming targeting WLWH, however, are largely focused on risk behaviours (such as condom use) rather than broader sex-positive considerations of sexual intimacy, well-being and satisfaction [13,14]. Moreover, prevailing concern about individual-level behaviours ignores the broader clinical, legal and social factors that regulate the sexual lives and rights of WLWH [15,16].

The criminalization of HIV non-disclosure [17] and “Treatment as Prevention” (TasP) [18] are two legal and public health strategies aimed at preventing HIV transmission that have emerged in many global settings, including Canada [11,19–21]. While both initiatives place sexual activity of PLWH and “Positive Prevention” (HIV prevention strategies directed at PLWH) at the centre of their efforts, they are theoretically opposing and deviate from the “shared responsibility” messaging for HIV prevention endorsed by the Global Network of People Living with HIV (GNP+), the Canadian AIDS Society and other international agencies [22,23]. Growing evidence demonstrates that early and sustained use of ART for greater than six months with viral suppression generates a very low risk of HIV transmission during condomless sex between HIV sero-discordant sexual partners, with recent studies reporting a transmission risk approaching zero [24–26]. These findings have supported the implementation of TasP initiatives in a number of global jurisdictions [20,27,28], where PLWH are monitored along a care cascade from HIV diagnosis to linkage and retention in care, and initiation and adherence to ART to achieve sustained viral suppression [29,30].

Despite the evidence and growing optimism about what it means to live with HIV, in terms of improved quality of life, clinical health and lowered transmission risks, Canada has among the most aggressive judicial approaches to prevent perceived sexual exposure to HIV through the criminalization of HIV non-disclosure [19,21]. In October 2012, the Supreme Court of Canada ruled that PLWH are legally required to disclose their HIV status to sexual partners prior to sexual activity that poses a “realistic possibility” of HIV transmission [17,31]. The Supreme Court defined realistic possibility as any sexual activity without the use of a condom and without a low HIV plasma viral load (defined by the court as VL<1500 copies/mL). PLWH who fail to meet both criteria and do not disclose their HIV status to sexual partners risk a criminal charge of aggravated sexual assault. If convicted, this charge results in jail time with a maximum sentence of life imprisonment and mandatory listing on a national Sexual Offender Registry. The inconsistency between legal definitions of the “realistic possibility” of HIV transmission and contemporary scientific assessments of HIV transmission risk and prognosis detract from rights-based approaches to improving the sexual health of WLWH and propagate misconceptions about the sexual and reproductive realities of living with HIV.

For many WLWH around the world, such public health and legal HIV prevention strategies have introduced increased surveillance (including monitoring VL, HIV disclosure and condom use) and present attendant consequences to their sexual lives. Importantly, expectations of HIV status disclosure exist within a context of widespread HIV-related fear, violence, and stigma and discrimination, which when combined with gender and relationship power inequities, disproportionately compromise WLWH's navigation of intimate relationships [32–35]. Together, these social factors mute conversations about sexuality and HIV, deny risks that WLWH face with forced disclosure and/or condom use, compromise opportunities for safe disclosure and sexual relationships for WLWH and reduce the willingness and agency of WLWH to seek out critical health services [36]. In Canada and elsewhere, increasing use of ART has not substantially alleviated the presence or impact of stigma and discrimination [37] despite attempts to normalize HIV through initiatives under the TasP umbrella [38,39].

In response to growing social, legal and public health surveillance, WLWH may adopt various strategies to protect and navigate their sexual lives, including the avoidance of sexual and romantic partnerships. Indeed, previous research has suggested that WLWH may forgo sexual activity to avoid expectations and risks of HIV disclosure to sexual partners [32–34]. To understand drivers of sexual activity decision-making among WLWH in Canada, we measured the prevalence of sexual inactivity and sexual satisfaction among a cohort of WLWH and assessed demographic, HIV clinical and socio-structural correlates of sexual inactivity.

## Methods

### Study setting

In Canada, approximately 16,600 women were living with HIV in 2011 [40], of whom 81% lived in one of the three provinces: British Columbia (BC), Ontario or Quebec.

### Study design

Baseline cross-sectional data were analyzed for WLWH enrolled in the Canadian HIV Women's Sexual and Reproductive Health Cohort Study (CHIWOS; www.chiwos.ca), a large, multisite, longitudinal, community-based research (CBR) project conducted by, with and for WLWH in BC, Ontario and Quebec (full cohort: *n*=1427). The primary objective of CHIWOS is to assess the prevalence, barriers and facilitators to use of women-centred HIV care, and the impact of such patterns of use on health outcomes. CHIWOS is grounded in Critical Feminist theory [41] and CBR principles, and guided by a Social Determinants of Women's Health framework [42,43] (described in detail elsewhere [44]). Consistent with CBR methodology, WLWH and allied clinicians, researchers, community partners, social and public health service delivery personnel, and policy-makers were involved in all stages of this research.

### Study population and recruitment

This analysis includes participants enrolled in CHIWOS between 27 August 2013 and 13 March 2015. WLWH aged ≥16 years from one of the three enrolling study provinces were eligible to participate. CHIWOS is inclusive to self-identified trans and cis gendered women, two-spirited women, gender queer and women of other gender identities. Given the importance of including young women's voices in the study, an exemption was sought and approved such that parental/guardian consent for women aged 16–18 years was not required.

WLWH were recruited for participation through peer word-of-mouth, HIV clinics in large and small communities across the three provinces, AIDS Service Organizations (ASOs), non-HIV community-based organizations (e.g. immigration and refugee services, women's shelters, harm reduction and sex worker support services), the networks of our national Steering Committee and three provincial Community Advisory Boards, and online methods (e.g. Listservs for WLWH, and our study website (www.chiwos.ca), Facebook page (www.facebook.com/CHIWOS) and Twitter feed (www.twitter.com/CHIWOSresearch).

Consistent with data showing an over-representation of Aboriginal and African, Caribbean and Black (ACB) women among WLWH in Canada, targeted efforts were made to recruit Aboriginal and ACB participants. These efforts included hiring a diverse national team of 38 WLWH as Peer Research Associates (PRAs), including 6 Aboriginal and 14 ACB PRAs, and the creation of a CHIWOS Aboriginal Advisory Board whose members assisted and advised on community outreach and recruitment.

Overall, 1355 women were enrolled in CHIWOS at time of analysis. For this analysis, 142 (10%) women were excluded, including 84 who requested to skip the Sexual Health section of the survey, 7 who reported a live birth at or less than six months prior to interview, 40 who reported never having had consensual sex, and 13 who were missing data on recent consensual sex (the primary outcome), yielding an analytic sample of 1213 WLWH. Given our objective to measure intentional sexual activity, women who experienced non-consensual sex in the six months prior to interview without report of consensual sexual activity, were not considered sexually active.

### Data collection

All participants completed a structured, online questionnaire (supported by FluidSurveys™ software) at baseline (study enrolment), administered by PRAs (WLWH hired as members of the study team). PRAs underwent extensive training in research ethics, CBR, consenting, administering questionnaires, social positioning, self-care and support for participants [45]. Questionnaires were conducted in English or French and administered in-person in a confidential setting at collaborating HIV clinics, ASOs or community organizations, or in women's homes. For some participants in rural or remote areas, questionnaires were administered via phone or Skype. Median survey completion time was 89 minutes [IQR: 71, 115] and participants received a $50 honorarium for their participation, regardless of whether all questions or sections of the questionnaire were completed.

### Measures

A national team of experts in women's health and HIV contributed to the development of the CHIWOS questionnaire, which was designed to maximize psychometric validity and reliability [46] and collected information on socio-demographics, HIV medical history and clinical care, experiences of violence and stigma and discrimination, and sexual, reproductive, women's and emotional health outcomes. Given the sensitive nature of questions about sexual health and violence, participants were given the option of completing these survey sections with the PRA, independently, or skipping the sections entirely.

#### Primary outcomes: sexual inactivity and sexual satisfaction

The primary outcome was recent sexual inactivity (vs. sexual activity) defined as no (vs. any) consensual sex (oral or penetrative) in the six months prior to interview.

Among all women, regardless of recent sexual activity or inactivity, sexual satisfaction was assessed using a five-point Likert scale item from the Sexual Satisfaction Scale for Women (SSS-W) [47]: “Overall, how satisfactory or unsatisfactory is your present sex life.” Responses were dichotomized into satisfactory (“Completely/Very/Reasonably satisfactory”) vs. unsatisfactory (“Not very/Not at all satisfactory”).

#### Covariates

Covariates of sexual inactivity included socio-demographic characteristics (i.e. age, education, gender identity, sexual orientation, ethnicity, annual household income, relationship status, and number of children); self-reported clinical HIV history and outcomes, including years living with HIV, receipt of HIV medical care in past year (yes vs. no), receipt of ART in past year (yes vs. no), most recent VL (undetectable (<40 copies/mL) vs. detectable ≥40 copies/mL, shown to have a high positive predictive value [48]), most recent CD4 cell count (<200 cells/mm^3^ vs. 200–500 cells/mm^3^ vs. >500 cells/mm^3^), as well as having ever discussed the impact of VL on risk of HIV transmission with a healthcare provider (yes vs. no) and perceptions of how ART changes personal risk of transmitting HIV (lowers the risk of transmission vs increases the risk of transmission or makes little difference); the SF-12 physical and mental health-related quality of life summary scores (scored on a 0–100 scale, where a higher score indicates better health) [49,50]; depression (measured using the 10-item Center for Epidemiologic Studies Depression Scale (CES-D 10), scored on a 0–30 scale, with higher scores indicating greater depression symptomology and a cutoff score of ≥10 considered indicative of “probable depression”) [51,52]; and HIV-related stigma measured using the 10-item HIV Stigma Scale (HSS) [53,54]. Scores for the HSS range from 0 to 100, with higher scores indicating higher stigma. Scores greater or equal to the scale median were considered “high HIV-related stigma” vs. “low HIV-related stigma.”

Among sexually active women, we describe types of sexual partnerships and knowledge about HIV status. In the survey, “Regular Sexual Partners” were defined to include, but not limited to, “spouses, common law partners, long term relationships, friends with benefits, or partners seen on and off for some time.” “Casual Sexual Partners” were defined to include, but not limited to, “serious sexual relationships that have recently begun, new sexual relationships that exist but you're not sure about, chance sexual encounters, or one night stands.”

### Data analysis

Descriptive statistics (median Interquartile Range [IQR]) for continuous variables and n (%) for categorical variables) were used to characterize baseline distributions of study variables. Baseline differences between sexually inactive and sexually active women were compared using Wilcoxon rank sum test for continuous variables and Pearson χ^2^ or Fisher's exact test for categorical variables.

We fit a multivariable logistic regression explanatory model to the data to examine covariates of sexual inactivity. After testing normality assumptions and collinearity, variables with a significant association with sexual inactivity in bivariate analyses (at *p*<0.20) were considered for the full model to obtain the relative contribution of each covariate. Model selection was achieved by minimizing the Akaike Information Criterion while maintaining Type III *p*-values for covariates below 0.20 [55]. All statistical tests were two-sided and were considered statistically significant at α=0.05. Data were analyzed using SAS version 9.3 (SAS Institute, Inc., Cary, NC).

### Ethical statement

All participants provided written, voluntary informed consent (or oral consent with a study team member present as a witness for surveys conducted by phone or Skype) at enrolment in the survey phase of the CHIWOS study. Ethical approval for all study procedures was provided by the Research Ethics Boards of Simon Fraser University, University of British Columbia/Providence Health, Women's College Hospital and McGill University Health Centre.

## Results

### Baseline characteristics

Of 1213 participants, 26% were from BC, 50% from Ontario and 24% from Quebec. Median age was 43 years [IQR: 35, 50], 95% identified as cis gender women and 5% identified as trans women, two-spirited or gender queer. Eighty-seven percent were heterosexual and 13% identified as LGBTQ (Lesbian, Gay, Bi-Sexual, Two-spirited, or Queer). Nearly one-quarter (23%) identified as Aboriginal, 28% ACB, 41% White and 8% other ethnicities. Sixty-three percent had an annual household income <$20,000 CAD, and 34% were married, common law or in a relationship.

Median years living with HIV was 10.8 [IQR: 5.9, 16.8], 94% reported receiving HIV medical care in the past year and 82% were currently taking ART. By self-report, 77% had undetectable VL and 50% had CD4 cell count >500 cells/mm^3^ at most recent visit. Sixty-nine percent of participants reported discussing the impact of VL on HIV transmission risk with their healthcare provider, and overall, 81% perceived that use of antiretrovirals lowers HIV transmission risk. Median SF-12 physical and mental health summary scores were 48 [IQR: 33, 56] and 42 [IQR: 31, 52], respectively. Median HIV stigma score was 60 [IQR: 50, 73] with 48% classified as experiencing high (vs. low) HIV-related stigma. Median depression symptom severity score (based on CES-D) was 9 [IQR: 4, 15], with 49% meeting the criteria for probable depression (Table 1).

**Table 1:** Baseline characteristics of women living with HIV enrolled in CHIWOS overall and by sexual activity (*n*=1213)

	Overall (*n*=1213)		Sexually active (*n*=618)	Sexually inactive (*n*=595)	
			
Characteristic	*n* (%) or median [IQR]	Total *n*	*n* (%) or median [IQR]		*p*-value
Median age [IQR] (years)	43 [35, 50]	1213	40 [33, 46]	46 [38, 54]	<0.001
Age category (years)					
16 to <30	114 (9.4)	1213	83 (13)	31 (5.2)	<0.001
30 to <40	366 (30)		218 (35)	148 (25)	
40 to <50	392 (32)		215 (35)	177 (30)	
50+	341 (28)		102 (17)	239 (40)	
Province					
British Columbia (BC)	317 (26)	1213	172 (28)	145 (24)	0.009
Ontario	607 (50)		283 (46)	324 (55)	
Quebec	289 (24)		163 (26)	126 (21)	
Gender identity^a^					
cis gender woman	1157 (95)	1213	581 (94)	576 (97)	0.02
Trans woman, two-spirited, gender queer, or other gender	56 (5)		37 (6)	19 (3)	
Sexual orientation^b^					
Heterosexual	1050 (87)	1209	525 (85)	525 (88)	0.121
LGBTQ	159 (13)		90 (15)	69 (12)	
Ethnicity					
Aboriginal	277 (23)	1213	142 (23)	135 (23)	0.391
African, Caribbean and Black Canadian (ACB)	338 (28)		164 (27)	174 (29)	
White	502 (41)		268 (43)	234 (39)	
Other ethnicities	96 (8)		44 (7.1)	52 (8.7)	
Education					
<High school	180 (15)	1207	93 (15)	87 (15)	0.798
≥High school	1027 (85)		520 (85)	507 (85)	
Annual household income (CAD)					
≥$20,000	419 (35)	1213	242 (39)	177 (30)	<0.001
<$20,000	759 (63)		353 (57)	406 (68)	
Don't know/prefer not to answer	35 (3)		23 (3.7)	12 (2.0)	
Relationship status					
Single/separated/divorced/widowed	802 (66)	1207	306 (50)	496 (84)	<0.001
Married/common law/in a relationship	405 (34)		307 (50)	98 (17)	
Number of children					
0	372 (32)	1162	175 (30)	197 (34)	0.204
1–3	630 (54)		332 (57)	298 (52)	
4+	160 (14)		78 (13)	82 (14)	
Median time living with HIV [IQR] (years)	10.8 [5.9, 16.8]	1180	10.1 [5.6, 15.8]	11.7 [6.2, 17.6]	0.004
Received HIV medical care in the past year					
Yes	1139 (94)	1212	574 (93)	565 (95)	0.102
No	73 (6.0)		44 (7.1)	29 (4.9)	
Currently taking ART					
Yes	999 (82)	1213	500 (81)	499 (84)	0.176
No	214 (18)		118 (19)	96 (16)	
Current plasma viral load (self-reported)					
Detectable (≥40 copies/mL)	173 (14)	1213	91 (15)	82 (14)	0.869
Undetectable (<40 copies/mL)	939 (77)		477 (77)	462 (78)	
Don't know/never received results	101 (8.3)		50 (8.1)	51 (8.6)	
Current CD4 cell count (self-reported)					
<200 cells/mm^3^	66 (5.5)	1211	34 (5.5)	32 (5.4)	0.597
200–500 cells/mm^3^	329 (27)		166 (27)	163 (28)	
>500 cells/mm^3^	606 (50)		302 (49)	304 (51)	
Don't know/never received results	210 (17)		116 (19)	94 (16)	
Discussed VL on HIV transmission risk with healthcare provider					
Yes	826 (69)	1201	461 (75)	365 (62)	<0.001
No	375 (31)		151 (25)	224 (38)	
Perception of how ART changes HIV transmission risk					
Lowers risk of transmission	979 (81)	1213	509 (82)	470 (79)	0.214
Increases risk of transmission or makes little difference	130 (11)		57 (9.2)	73 (12)	
Don't know	104 (8.6)		52 (8.4)	52 (8.7)	
Median health-related quality of life score [IQR]					
Physical health summary score	48 [33, 56]	1206	50 [36, 56]	46 [32, 55]	0.004
Mental health summary score	42 [31, 52]	1206	42 [31, 52]	43 [32, 53]	0.537
HIV stigma scale					
High HIV-related stigma	575 (48)	1200	280 (46)	295 (50)	0.155
Low HIV-related stigma	625 (52)		330 (54)	295 (50)	
Satisfaction with present sex life					
Completely, very or reasonably satisfied	777 (64)	1213	488 (79)	289 (49)	<0.001
Not very or not at all satisfied	347 (29)		115 (19)	232 (39)	
Prefer not to answer	89 (7.3)		15 (2.4)	74 (12)	
Probable depression (CESD-10)					
No (score<10)	600 (51)	1173	330 (55)	270 (47)	0.007
Yes (score ≥10)	573 (49)		270 (45)	303 (53)	

Notes:

a Gender identity was self-reported as cis or trans gender woman, two-spirited, gender queer or an ‘other’ gender identity. Given small numbers, we grouped participants self-identifying as trans gender, two-spirited, gender queer or ‘other’ gender for this analysis. Of the 56 women included in this grouped category, 48 (86%) identified as transgender women.

b LGBTQ includes participants who identify as Lesbian, Gay, Bi-sexual, Two-spirited or Queer; CAD=Canadian dollars; ART=antiretroviral therapy; VL=viral load.

### Sexual inactivity, sexual satisfaction and summary of sexual partnerships

Forty-nine percent (49%) of participants reported being sexually inactive in the six months prior to baseline interview. Sixty-four percent (64%) of women reported being satisfied with their current sex lives, including 49% of sexually inactive women and 79% of sexually active women (*p*<0.001).

Of sexually active WLWH (*n*=618), 81% had one and 6% had two or more regular sexual partners in the previous six months. Of those with at least one regular sexual partner (*n*=543), 27% reported that their primary sexual partner was HIV-positive, 64% reported that the partner was HIV-negative and for 9% the partner's HIV status was unknown. Ninety-one percent (91%) reported that their current or most recent regular partner knew the participant's HIV status at their last sexual encounter. One-fifth (21%) of all sexually active participants reported having a casual sexual partner in the previous six months.

### Correlates of sexual inactivity

In unadjusted analyses, sexual inactivity was associated (at *p*<0.20) with older age, gender identity, sexual orientation, household income <$20,000 per year, not being in a martial or committed relationship, longer time living with HIV, current ART use, poorer physical health, high HIV-related stigma scores, probable depression, sexual dissatisfaction and having discussed the role of VL on HIV transmission risk with a healthcare provider (Table 2).

**Table 2:** Unadjusted and adjusted odds ratios for correlates of sexual inactivity in the previous six months among women living with HIV enrolled in CHIWOS

	Sexually inactive vs. Sexually active over the last six months	
	
Characteristics	Unadjusted OR (95% CI)	Adjusted OR (95% CI)
Age, per one year increase	1.06 (1.05–1.08)	1.06 (1.05–1.08)
Sexual orientation		
Heterosexual	1.0	
LGBTQ	0.75 (0.54–1.05)	
Annual household income (CAD)		
≥$20,000	1.0	1.0
<$20,000	1.57 (1.24–2.0)	1.44 (1.08–1.92)
Relationship status		
Married/common law/in a relationship	1.0	1.0
Single/separated/divorced/widowed	5.00 (3.84–6.67)	4.34 (3.13–5.88)
Currently taking ART		
Yes	1.0	
No	0.82 (0.61–1.10)	
Discussed VL on HIV transmission risk with healthcare provider		
Yes	1.0	1.0
No	1.87 (1.46–2.4)	1.57 (1.16–2.11)
HIV-related stigma		
Low HIV-related stigma	1.0	1.0
High HIV-related stigma	1.18 (0.94–1.47)	1.81 (1.09–3.03)
Probable depression (CESD-10)		
No (score <10)	1.0	
Yes (score ≥10)	1.37 (1.09–1.73)	
Physical Health Summary score	0.99 (0.98–0.99)	
Satisfaction with present sex life		
Completely, very, or reasonably satisfied	1.0	
Not very or Not at all satisfied	3.41 (2.61–4.45)	
Prefer not to answer	8.33 (4.69–14.8)	

Note: Model adjusted for Province of residence; LGBTQ includes participants who identify as Lesbian, Gay, Bi-sexual, Two-spirited or Queer; CAD=Canadian dollars; ART=antiretroviral therapy; VL=viral load.

In the logistic regression model, sexually inactive women had significantly higher adjusted odds of being older (AOR=1.06 per year increase in age; 95% CI=1.05–1.08), not being in a marital or committed relationship (AOR=4.34; 95% CI=3.13–5.88), having an annual household income below $20,000 CAD (AOR=1.44; 95% CI: 1.08–1.92), and reporting high (vs. low) HIV-related stigma (AOR=1.81; 95% CI=1.10–3.03). No independent association was found with current ART use, undetectable plasma HIV VL or CD4>500 cells/mL. However, sexually inactive women were significantly more likely to report not having discussed the role of VL on decreasing HIV transmission risk with a healthcare provider (AOR=1.57; 95% CI: 1.16–2.11; Table 2).

## Discussion

This is the first multisite cohort study to evaluate sexual inactivity among WLWH in Canada in the modern era of TasP and HIV criminalization. Nearly half of WLWH in this Canadian cohort reported being sexually inactive and over two-thirds report being satisfied with their sexual lives. Other studies have similarly shown that sexual inactivity is common among PLWH, particularly women. The Women's Interagency HIV Study (WIHS), an on-going cohort study of WLWH in the United States (US), found that 35% of WLWH reported no vaginal, oral or anal sex over a six-month time period compared with 23% of HIV-negative women [56]. Other US research has found that WLWH are significantly more likely to be sexually inactive (34%) compared with HIV-positive gay and bisexual men (28%) [57]. Similar results were seen in a study by Bogart *et al*. [58], where a higher proportion of WLWH (18%) were deliberately abstinent compared to gay and bisexual men with HIV (11%). The prevalence of sexual inactivity (49%) in our study was notably higher than estimates from other HIV cohorts. Some of this difference may be explained by the older median age of WLWH enrolled in CHIWOS compared with other referenced cohorts and the evidence that sexual activity and satisfaction decline with increasing age [59,60]; and shown in Table 1. Some difference may also be explained by our distinction between and exclusion of non-consensual sex in our definition of sexual activity. However, the remaining difference may be explained by a high reported prevalence of HIV stigma and the context of surveillance for WLWH in the Canadian setting [61]. Our estimates of sexual inactivity are also significantly higher than those reported in the general population (10–14%) [62], echoing previous research that an HIV diagnosis impacts the sexual health of women [63] even several years after the initial diagnosis [64].

The association between sexual inactivity and high HIV-related stigma and sexual dissatisfaction but not ART use or viral suppression suggests that WLWH face challenges navigating healthy, satisfying and safe sexual lives, despite good treatment outcomes. This suggests that good treatment outcomes alone do not lead to higher likelihood of sexual activity or eliminate prevailing socio-structural barriers to sexual health for WLWH.

HIV-related stigma and discrimination are critical barriers to cultivating loving, intimate relationships, facilitating HIV status disclosure and engaging with healthcare for WLWH [65,66]. The increasingly strict use of the criminal law for HIV non-disclosure against PLWH in Canada presents an additional form of stigmatization [67] and additional challenges for WLWH initiating sexual relationships and navigating HIV disclosure to sexual partners. This judicial approach shifts the responsibility for condom use and the burden of proof onto PLWH, introducing the potential for false accusations of HIV non-disclosure by sexual partners and concerns of secondary disclosure within the wider community [68,69]. Observations that the sexuality of PLWH is being policed [70] and reports of discriminating medical surveillance in the reproductive healthcare setting [61] raise concerns that the sexual rights of WLWH are being unjustly compromised in the current legal climate. While we are unable to directly assess the effect of criminalization of HIV non-disclosure on sexual activity within this study, qualitative studies in Canada have found that fear, anxiety and uncertainty related to the current legal obligation to disclose HIV serostatus to sexual partners have resulted in some PLWH abstaining from sexual activity altogether [70]. Forgoing sexual activity removes expectations and risks of HIV disclosure to sexual partners, difficulties negotiating condom use and risks of sexual HIV transmission. For some WLWH, sexual abstinence may also be a response to fear and threats of rejection by sexual partners, secondary disclosure, relationship dissolution, violence and social isolation following disclosure of HIV status [32,58,71,72].

Despite the high prevalence of sexual inactivity in our cohort, a large proportion of women overall report being satisfied with their current sex lives (64%). While satisfaction differs by sexual activity, it is higher overall than expected based on previous research [60]. Among sexually inactive women, half report being satisfied with their present sex lives, suggesting that sexual abstinence may be deliberate or intentional, not simply circumstantial. However, sexually inactive women report higher HIV-related stigma. This suggests that HIV-related stigma plays an important role in sexual decision-making, expression and lives of WLWH. Furthermore, WLWH experiencing high HIV-related stigma may be actively choosing sexual inactivity as a strategy to avoid having to navigate disclosure and risk stigma and discrimination in initiating sexual partnerships, and for many women this is a satisfactory approach to their sexual lives.

Interventions are required to reframe the approach to sexual health among WLWH. A component of this reframing is a movement towards models that advocate for Positive Health, Dignity and Prevention frameworks [23,73], which place the person living with HIV at the centre of their health, care and well-being, well beyond a role in “positive prevention” of on-going transmission of HIV. For example, the Au-delà du VIH: être femme Plurielle [Beyond HIV: being a woman in its multiple meanings] program in Quebec is grounded in an empowerment approach to sexual health for WLWH, and supports the capacity of WLWH to respond to their sexual needs and mobilize available resources to gain control over their sexual lives. This, and other similar interventions, recognize WLWH as sexual beings with sexual rights and aim to overcome beliefs and taboos of female sexuality and desire [74].

Limitations to this study are acknowledged. First, cross-sectional analyses preclude determination of causality between correlates and sexual inactivity, and reverse causality is possible (e.g. sexual inactivity leading to depression). Second, there is risk of reporting bias whereby women may underreport sexual activity because of prevailing stigma and discrimination against sexually active WLWH. However, surveys were administered by PRAs, a diverse group of WLWH with identities and life experiences in common with participants, which may have decreased the extent of social desirability bias in reports about sexual activity (with <10% of participants completing the sexual health section independently). Third, we are unable to report whether sexual inactivity represents deliberate or intentional abstinence for sex or a lack of opportunity for sex; however, this difference will be explored in the CHIWOS 18-month follow-up questionnaire. Finally, several important psychosocial aspects of sexual inactivity, including sensation seeking, desire and motivation, as well as knowledge and perceived effect of criminalization of HIV non-disclosure on sexual activity, were not directly assessed in this study, and are likely to be important explanatory pathways to understanding sexual inactivity among WLWH. These pathways will also be assessed in the follow-up questionnaire.

## Conclusions

A satisfying sexual life is a critical component of health and well-being for all people, including WLWH. The observed high rate of sexual inactivity coupled with associations with HIV-related stigma and sexual dissatisfaction underscores a need to revisit the narrative about sexual activity among WLWH. This narrative has focused heavily on HIV risk reduction approaches and now increasingly on the public health importance of viral suppression and the legal test for HIV non-disclosure, but much less has been said about healthy sexuality. A transitioning of the approach to healthy sexuality among WLWH requires that we finally erase the current view of WLWH as “vectors, vessels and victims” of HIV [75], rather as empowered individuals with agency and deserving of intimate relationships. These findings underscore an urgent need for public health and socio-structural interventions to de-stigmatize HIV, support safe disclosure and re-appropriate the sexual rights of WLWH.
